# Dysregulation of Autonomic Nervous System in Chagas’ Heart Disease Is Associated with Altered Adipocytokines Levels

**DOI:** 10.1371/journal.pone.0131447

**Published:** 2015-07-06

**Authors:** João Marcos Barbosa-Ferreira, Charles Mady, Barbara Maria Ianni, Heno Ferreira Lopes, Felix José Alvarez Ramires, Vera Maria Cury Salemi, Cesar José Grupi, Denise Tessariol Hachul, Fábio Fernandes

**Affiliations:** 1 Cardiomyopathy Unit of the Heart Institute (InCor), do Hospital das Clínicas da Faculdade de Medicina da Universidade de São Paulo, São Paulo, Brazil; 2 Hypertension Unit of the Heart Institute (InCor), do Hospital das Clínicas da Faculdade de Medicina da Universidade de São Paulo, São Paulo, Brazil; 3 Universidade Nove de Julho—UNINOVE, São Paulo, Brazil; 4 Electrocardiology Unit of the Heart Institute (InCor), do Hospital das Clínicas da Faculdade de Medicina da Universidade de São Paulo, São Paulo, Brazil; 5 Clinical Arrhythmia Unit of the Heart Institute (InCor), do Hospital das Clínicas da Faculdade de Medicina da Universidade de São Paulo, São Paulo, Brazil; Albert Einstein College of Medicine, UNITED STATES

## Abstract

**Background:**

Chagas disease (CD) induces autonomic dysfunction and inflammatory activity, which may promote metabolic abnormalities. We studied metabolism and his correlation with Autonomic Nervous System (ANS) and inflammation in CD.

**Methods and Results:**

Sixty subjects were divided into 4 groups: control group (CG), IF (indeterminate form) group; ECG group (ECG abnormalities and normal left ventricular systolic function), and LVD group (left ventricular sistolic dysfunction). Levels of adiponectin, leptin, insulin, interleukin-6 (IL-6), and tumor necrosis factor-alpha (TNF-alpha) were assayed in serum samples by ELISA. ANS was assessed by heart rate variability in frequency domain in 24-hour Holter and postural tilt test (rest and orthostatic position). High frequency (HFr) component values were used to estimate parasympathetic activity and low frequency (LFr) component, sympathetic activity. Analyzes were made of the correlations of each of the metabolic parameters (leptin and adiponectin) with the inflammatory cytokines (interleukin-6 and TNF- alpha) and with the ANS assessment measurements. No significant differences were observed in leptin and insulin levels. Adiponectin was higher in ECG and LVD groups: [CG = 4766.5 (5529.5), IF = 4003.5 (2482.5), ECG = 8376.5 (8388.5), LVD = 8798 (4188.0) ng/mL, p<0.001)]. IL-6 and TNF-alpha were higher in LVD group: [IL-6: CG = 1.85 (6.41); IF = 1.58 (1.91); ECG = 1.0 (1.57); LVD= 31.44 (72.19) pg/ml; p = 0.001. TNF-alpha: CG = 22.57 (88.2); IF = 19.31 (33.16); ECG = 12.45 (3.07); LVD = 75.15 (278.57) pg/ml; p = 0.04]. Adiponectin levels had a positive association with the HFr component (r = 0.539; p = 0.038) and an inverse association with the LFr component (r = - 0.539; p = 0.038) in ECG group. Leptin levels had a negative association with the HFr component (r= - 0.632; p = 0.011) and a positive association with the LFr component (r = 0.632; p = 0.011) in LVD group.

**Conclusions:**

We found increased adiponectin levels in Chagas’ heart disease with systolic dysfunction and in patients with ECG abnormalities and normal systolic function at rest. Adipocytokines levels (adiponectin and leptin) were associated with ANS parameters in Chagas’ heart disease.

## Introduction

Chagas’ disease (CD) occurs from the southern United States to Patagonia and affects around 8 million people in Latin America [1]. Moreover, due to the intensification of the migratory flow, CD is becoming more relevant in nonendemic countries, such as the United States, Canada, some European countries, Japan, and Australia. In the United States, it is estimated that 300 thousand legal immigrants may be infected with the disease. Spain has the second largest prevalence with around 40 to 60 thousand infected immigrants [2]. The natural history of Chagas’ disease is summarized in the acute and chronic phases[3]. In the chronic phase, about 70% of the patients have no symptoms and routine examinations do not show any abnormalities. This stage is called indeterminate form (IF). The patients of IF form of Chagas’ disease, in general, have a very good prognosis. Survival in this group appears comparable to that of the general population [4]. The remaining 30% have the chronic digestive and/or cardiac form, and 10% of these patients may progress to severe forms of heart disease. The progression to myocardial dysfunction represents the leading cause of morbidity and mortality in Chagas’ disease[3]. Therefore, investigations regarding the pathophysiology of the development and progression of cardiomyopathy are of fundamental importance in the proposed new therapies in an attempt to minimize morbidity and mortality.

Myocardial damage directly related to parasite persistence, immunologic mechanisms, microvascular disturbances, and autonomic dysfunction are involved in the pathophysiological mechanism of chagasic cardiomyopathy [5]. Cardiac dysautonomia is a well-established feature of Chagas disease, in which anatomic denervation and functional abnormalities have been extensively described. Neuronal depopulation occurs in cardiac parasympathetic ganglia in Chagas’ heart disease associated with scattered sympathetic denervation [6]. Several methods are currently available to evaluate autonomic function such as Valsalva maneuver, deep breathing, orthostatic test and heart rate variability in the time domain or in the frequency domain [5]. Previous studies suggest that autonomic dysfunction may precede left ventricular systolic dysfunction [7,8]. The mechanisms of Chagas’ cardiomyopathy can influence other pathophysiological pathways, such as metabolic impairment.

Recent research has led to a growing appreciation of the complexity of metabolic aspects of heart failure (HF) pathophysiology. Not only the myocardium, but also peripheral tissues and organs are affected by metabolic failure, resulting in a global imbalance between catabolic and anabolic signals. Metabolic feedback signals from muscle and fat actively contribute to disease progression [9]. The adipocytokines are bioactive mediators produced by adipose tissue. The main adipocytokines are adiponectin and leptin. Adiponectin has beneficial anti-inflammatory and anti-atherogenic effects as well as insulin-sensitizing action [10–14]. However, the role of adiponectin in cardiovascular disease is still a controversy. Previous studies have demonstrated that adiponectin is increased in systolic HF patients, even predicting morbidity and mortality [15,16]. Leptin promotes pro-inflammatory and pro-thrombotic activities, neointimal proliferation, endothelial dysfunction and induction of insulin resistance [17,18]. In CD patients, we have previously found reduced leptin levels in Chagas’ cardiomyopathy when compared with a control group and other forms of CD [19]. Our speculation is that chagasic patients have altered sympathetic activity resulting in decreased leptin synthesis.

So, the basic hypothesis is that chronic Chagas’ cardiomyopathy may serve as a model in which severe inflammatory activity and early involvement of the autonomic nervous system (ANS) influence metabolism. Alterations in metabolism such as insulin resistance and the role of adipose tissue can lead to a systemic inflammatory state that contributes to vasculopathy and cardiovascular risk [14]. This two-way mechanism can bring chronic metabolic complications of HF to the fore and gradually shift its clinical presentation. The study of this association can promote emerging therapeutic concepts with specific metabolic targets. The aim of this study was to evaluate the metabolic parameters in the different forms of Chagas’ disease and its association with inflammatory activity and with measures of autonomic nervous system function.

## Materials and Methods

### Patient selection

Sixty subjects were divided into 4 groups: control group (CG), IF (indeterminate form) group of Chagas disease; ECG group (Chagas heart disease with ECG abnormalities and normal left ventricular systolic function), and LVD group (Chagas heart disease with left ventricular systolic dysfunction). The control group consisted of 15 healthy individuals. IF group comprised subjects with 2 positive serologic reactions for Chagas' disease and no cardiac involvement as defined by chest X-rays, 12-lead ECG, and 2-dimensional echocardiography. All patients had normal barium studies of the esophagus and the colon. ECG group consisted of patients with normal left ventricular (LV) systolic function showing right or left bundle-branch block, left anterior fascicular block, diffuse ST changes, ventricular premature beats that may be multiform or runs of non-sustained ventricular tachycardia registered on the ECG. LVD group, comprised patients with LV dysfunction demonstrated by a left ventricular ejection fraction less than 40% in echocardiography. All of these groups were matched according to sex, age (± 2 years intervals), and body mass index (± 1 kg/m^2^ intervals). The inclusion criteria were patients with 2 positive serologic reactions for Chagas' disease and age over 18 years. The exclusion criteria were myocardial infarction (evaluated by Q waves on an electrocardiogram (ECG) or segmental left ventricular (LV) dysfunction by 2-dimensional echocardiography), moderate or severe valvar heart disease (evaluated by clinical examination and by Doppler echocardiography), arterial hypertension, smoking, diabetes mellitus, current use of statins, atrial fibrillation, advanced atrioventricular block, pacemaker, thyroid diseases, chronic obstructive pulmonary disease, and heart failure functional classes III and IV by the New York Heart Association (NYHA) classification. All subjects signed a written consent form, and the Ethics Committee of the Heart Institute (InCor), University of São Paulo Medical School approved the study.

### Antropometric measurements

All subjects underwent anthropometric measurements such as weight, height, body mass index, and waist circumference. Bioelectrical impedance (BIA 450, Byodinamics, Seattle, USA) was used to assess percentage of body fat and fat mass.

### Echocardiographic study

Comprehensive transthoracic echocardiographic studies were performed with a Sequoia 512 ultrasound machine (Acuson, Montain View, CA USA). Two-dimensional guided M-mode measurements of the left ventricle (LV) in short axis view and LV, left atrium and right atrium end diastolic and end systolic volumes were taken from apical 4-chamber view. LV ejection fraction (LVEF) was calculated using a modified Simpson biplane method. LV regional wall motion was evaluated based on 17-segment model segmentation, and each segment was confirmed in multiple views as follows: normal or hyperkinesis, hypokinesis, akinesis, dyskinesis, and aneurysmal. Diastolic function was assessed from pulsed-wave Doppler of the transmitral inflow velocities, with the sample volume at the mitral valve leaflet tips, and from tissue Doppler imaging of the septal and lateral mitral annulus, both at apical 4-chamber view. Mitral, tricuspid, aortic, and pulmonary regurgitations were qualitatively evaluated.

### Laboratory measurements

Fasting glucose, total cholesterol, HDL-cholesterol, LDL-cholesterol and tryglicerides were measured using standard assays.

The subjects underwent serum adiponectin, insulin, leptin, interleukin-6 (IL-6), and tumor necrosis factor-alpha (TNF-alpha) measurements by ELISA. Serum insulin was measured with a commercially available kit (Millipore, St. Charles, Missouri, USA). The sensitivity of the kit was 1 μU/mL, and the reference interval was from 31 to 65 μU/ml. The measurements were made in duplicate with a coefficient of variation of 4.3%. Serum leptin was measured with a commercially available kit (Millipore, St. Charles, Missouri, USA). The sensitivity of the kit was 0.78 ng/mL, and the reference interval was from 12.9 to 26.8 ng/mL. The measurements were made in duplicate with a coefficient of variation of 7.3%. Serum adiponectin levels were measured with a commercially available kit (Millipore, St. Charles, Missouri, USA). The sensitivity of the kit was 100 ng/mL, and the reference interval was from 7000 to 14,500 ng/mL. The measurements were made in duplicate with a coefficient of variation of 3.1%. Interleukin-6 was measured with a commercially available kit (USCNK, Life Science Inc., Wuhan, China). The sensitivity of the kit was 5.6 pg/mL, and the reference interval was from 75 to 175 pg/mL. The measurements were made in duplicate with a coefficient of variation of 10.3%. Tumor necrosis factor-alpha was measured with a commercially available kit (USCNK, Life Science Inc., Wuhan, China). The sensitivity of the kit was 5.9 ng/mL, and the reference interval was from 150 to 175 pg/mL. The measurements were made in duplicate with a coefficient of variation of 6.4%.

### Evaluation of autonomic nervous system function

The autonomic nervous system (ANS) function was evaluated by heart rate variability (HRV) in the frequency domain using Fast Fourier Transform model in 24-hour Holter and postural tilt test (rest and orthostatic position). The following indices were calculated: Total power, Low frequency (LFr) component in absolute values of power (ms^2^) and in normalized units (n.u.), High frequency (HFr) component in absolute values of power (ms^2^) and in normalized units (n.u.), and the LFr/HFr ratio. We also calculated the changes in LFr and HFr components from rest to orthostatic position. The increase of the LF component and the LFr/HFr ratio were interpreted as a predominance of sympathetic activity. The increase in the HFr component and the reduction in the LFr/HFr ratio were interpreted as a predominance of parasympathetic activity[20].

The following formula was used for the calculation of the LFr and HFr components in normalized units (n.u.):

- Component LFr or HFr in absolute values / (total power–very low frequency component) x 100

The patients were instructed not to take any stimulants, such as coffee, tea, soft drinks and alcoholic beverages, on the day prior to and on the day of the exams.

The “Task Force Monitor” (CNSystems Medizintechnik GmbH, Graz, Austria) was used for analysis during postural tilt testing. The five minutes immediately prior (rest in supine position) and the five minutes immediately after inclination (orthostatic position) were assessed. The mean of the spectral components obtained in the established times was calculated. Cardiac cycles with a variation greater than 25% to a previous one were excluded as a way of abolishing the consequent alterations to the ventricular and supraventricular extrasystoles. Only the readings with at least 85% sinus beats were assessed.

### Statistical analyses

Statistical analyses were performed using ANOVA test to evaluate differences in means among groups. If the homogeneity of the variances was not observed, the non-parametric Kruskal-Wallis test was used. When there is evidence of difference in at least one group, it was used Tukey tests for comparisons. Spearman coefficient was used for correlation analysis. We evaluated the correlations in each of the three groups of patients with Chagas disease separately (IF group, ECG group and LVD group). The correlations were made between each of the adipocytokines (adiponectin and leptin) with the inflammatory cytokines (interleukin-6 and TNF- α) and with the ANS assessment measurements (in normalized units). A P value < 0.05 was considered statistically significant.

## Results

### Clinical characteristics

There were no significant differences in age, weight, and body mass index among groups. The ejection fraction was significantly lower in LVD group. Systolic and diastolic blood pressure was lower in the LVD group compared to other three groups. The fat mass was lower in the LVD group, with statistical significance for the absolute values. The drugs used by the individuals of the LVD group were as follows: amiodarone in three patients (20%), beta-blockers in 15 patients (100%), angiotensin converting enzyme inhibitors in 13 patients (86.6%), angiotensin receptor blockers in two patients (13.3%), furosemide in nine patients (60%), spironolactone in 11 patients (73.3%) and digoxin in one patient (6.6%). Individuals from others groups did not use any drugs. Baseline physical and hemodynamic characteristics in Chagas' disease patients and healthy controls are displayed in Table 1.

**Table 1:** Clinical and hemodynamic characteristics of the studied population.

Variable	Control Group	IF Group	ECG Group	LVD Group	p value *
Age (years)	43.8 (± 7.43)	42.33 (± 7.30)	43.20 (± 6.14)	42.67 (± 6.72)	0.942
NYHA (I/II)	-	-	-	4/11	-
HR (bpm)	74.93 (± 7.00)	73.47 (± 9.20)	73.33 (± 8.20)	69.47 (± 10.91)	0.604
SBP (mmHg)	122.67 (± 7.04)	120.67 (± 9.61)	121.33 (± 9.90)	101.33 (± 9.90)**	<0.001
DBP (mmHg)	70.00 (± 7.56)	72.67 (± 7.99)	74.00 (± 7.37)	62.67 (± 5.94)**	0.001
BMI (kg/m^2^)	23.9 (± 1.20)	23.9 (± 1.39)	23.7 (± 1.20)	23.2 (± 1.50)	0.602
Weight (kg)	70.4 (± 8.50)	68.1 (± 10.00)	67.8 (± 8.50)	65.8 (± 8.80)	0.595
Fat mass (kg)	15.7 (± 4.40)	14.5 (± 5.80)	14.3 (± 6.20)	10.5 (± 3.30)**	0.015
Fat mass (%)	22.9 (± 5.50)	20.7 (± 8.10)	21.4 (± 9.20)	16.1 (± 5.00)	0.071
LVEF (%)	74.67 (± 4.61)	74.87 (± 6.02)	69.40 (± 8.97)	30.20 (± 5.76)**	0.049

Values expressed as mean (± SD)

* p values were calculated using ANOVA

** p<0.001 compared to control group using Tukey test

NYHA: New York Heart Association functional class; HR: heart rate; SBP: systolic blood pressure; DBP: diastolic blood pressure, BMI: body mass index; LVEF: left ventricular ejection fraction; kg: kilograms, %: percentage

### Laboratory measurements

There were no significant differences in fasting glucose, total cholesterol, HDL-cholesterol, LDL-cholesterol, tryglicerides, leptin, and insulin among groups. Adiponectin was significantly increased in ECG and LVD groups compared to IF and control groups (p<0.001). The levels of interleukin-6 (p = 0.001) and tumor necrosis factor-alpha (p = 0,04) were higher in the LVD group compared to the other three groups. The data from metabolic and inflammatory parameters are described in Table 2.

**Table 2:** Metabolic and inflammatory parameters.

Variable	Control Group	IF Group	ECG Group	LVD Group	p value *
Insulin (μU/mL)	3.41 (1.98)	4.31 (2.85)	4.3 (3.06)	4.58 (2.88)	0.901
Leptin (ng/mL)	3.42 (7.43)	3.03 (6.53)	5.56 (6.20)	2.86 (2.67)	0.626
Adiponectin (ng/mL)	4766.5 (5529.50)	4003.5 (2482.50)	8376.5 (8388.50)**	8798.0 (4188.00)**	<0.001
Interleukin-6 (pg/mL)	1.85 (6.41)	1.58 (1.91)	1.00 (1.57)	31.44 (72.19)**	0.001
TNF-alpha (pg/mL)	22.57 (88.20)	19.31 (33.16)	12.45 (3.07)	75.15 (278.57)**	0.040
Fasting glucose (mg/dL)	93.47 (± 14.41)	91.07 (± 9,04)	93.80 (± 6.07)	93.80 (± 8.18)	0.589
Total cholesterol (mg/dL)	190.00 (± 28.03)	186.10 (± 46.83)	190.60 (± 31.09)	193.70 (± 38.85)	0.956
LDL-cholesterol (mg/dL)	116.60 (± 25.14)	114.20 (± 42.00)	119.80 (± 25.03)	123.10 (± 34.38)	0.689
HDL-cholesterol (mg/dL)	53.07 (± 17.05)	48.47 (± 1.84)	45.67 (± 8.76)	49.53 (± 9.30)	0.571
Tryglicerides (mg/dL)	101.30 (± 69.56)	112.4 (± 63.61)	125.8 (± 68.89)	105.3 (± 81.80)	0.395

Values expressed as medians (interquartile range))

* p values were calculated using Kruskal-Wallis test

** p <0.05 compared to the control group using Tukey test

TNF-alpha: tumor necrosis factor alpha

### Evaluation of autonomic nervous system function

The results of ANS study suggest a greater impairment of sympathetic branch in LVD group and a balanced impairment of parasympathetic and sympathetic branchs in IF group and in ECG group. The data related to the 24-hour Holter and postural tilt test in supine (rest) and in orthostatic position are detailed in Tables 3, 4 and 5.

**Table 3:** Evaluation of autonomic nervous system indices using the 24-hour Holter.

Variable	Control Group	IF Group	ECG Group	LVD Group	p value *
Total power (ms^2^)	2091.41 (1102.40)	1181.28 (817.30)**	1202.53 (1316.20)	1178.97** (771.30)	0.03
LFr (ms^2^)	678.00 (524.70)	422.30 (316.70)	324.30 (442.00)	284.90** (316.60)	0.007
HFr (ms^2^)	170.00 (161.50)	63.50** (56.60)	94.40 (153.10)	58.80 (181.60)	0.008
LFr (n.u.)	75.60 (15.20)	86.20** (7.10)	81.60 (26.50)	75.00 (30.00)	0.022
HFr (n.u.)	24.30 (15.20)	13.70** (7.10)	18.30 (19.90)	24.90 (30.00)	0.016
LFr/HFr Ratio	3.10 (3.30)	6.20** (3.00)	4.40 (5.80)	3.00 (4.20)	0.028

Values expressed as medians (interquartile range)

* p values were calculated using Kruskal-Wallis test

** p <0.05 compared to control group using Tukey test

LFr: low frequency; HFr: high frequency; ms^2^: milliseconds squared; n.u.: normalized units.

**Table 4:** Evaluation of autonomic nervous system indices using the postural tilt test (supine position).

Variable	Control Group	IF Group	ECG Group	LVD Group	p value *
Total power (ms^2^)	2182.99 (1308.00)	945.9 (1551.00)	646.37** (589.02)	1073.10 (2308.01)	0.002
LFr (ms^2^)	560.71 (364.42)	264.61** (450.93)	104.75** (68.47)	178.46** (557.54)	0.001
HFr (ms^2^)	326.89 (178.71)	73.64** (157.00)	72.60** (280.00)	362.60 (1005.11)	< 0.001
LFr (n.u.)	68.74 (12.35)	70.78 (21.18)	63.19 (18.26)	27.68** (37.57)	0.022
HFr (n.u.)	31.01 (11.61)	29.21 (21.18)	36.80 (18.26)	72.31** (39.36)	0.023
LFr/HFr Ratio	2.19 (1.36)	2.42 (2.42)	1.71 (1.10)	0.38** (1.10)	< 0.001

Values expressed as medians (interquartile range)

* p values were calculated using Kruskal-Wallis test

** p <0.05 compared to control group using Tukey test

LFr: low frequency; HFr: high frequency; ms^2^: milliseconds squared; n.u.: normalized units.

**Table 5:** Evaluation of autonomic nervous system indices using the postural tilt test (orthostatic position).

Variable	Control Group	IF Group	ECG Group	LVD Group	p value *
Total power (ms^2^)	1891.20 (1325.30)	596.28** (558.82)	613.36** (896.44)	1001.77** (1122,88)	0.005
LFr (ms^2^)	666.73 (1018.90)	213.24** (262.26)	260.53** (356.76)	84.65** (394,67)	0.009
HFr (ms^2^)	154.75 (157.22)	39.18** (77.01)	98.02 (94.55)	329.95 (612,96)	< 0.001
LFr (n.u.)	83.21 (14.21)	82.37 (11.50)	77.80 (28.16)	35.97** (42.26)	0.007
HFr (n.u.)	16.78 (14.21)	17.62 (11.50)	22.19 (28.16)	64.02** (42.26)	0.006
LFr/HFr Ratio	4.95 (4.13)	4.67 (5.72)	3.50 (2.89)	0.56** (1.34)	0.019
Change LFr (n.u.)	21.58 (23.04)	14.14 (25.34)	8.26 (16.33)	4.93 (51.83)**	0,02
Change HFr (n.u.)	-37.11 (33.97)	-42.96 (46.60)	-25.80 (25.63)	-9,85 (18.18)**	< 0,001

Values expressed as medians (interquartile range)

* p values were calculated using Kruskal-Wallis test

** p <0.05 compared to control group using Tukey test

LFr: low frequency; HFr: high frequency; ms^2^: milliseconds squared; n.u.: normalized units.; Change LFr and HFr (n.u.): Change of each component from rest to orthostatic position in normalized units

### Correlations

There were significant correlations between adiponectin with some ANS assessment indexes in ECG group. Adiponectin level is associated negatively with the LFr component and positively with the HFr component in 24-hour Holter. No correlations between adiponectin and inflammatory cytokines were found. The values of correlations of adiponectin are shown in Table 6.

**Table 6:** Spearman rank correlation coefficients of adiponectin with autonomic nervous system indices and inflammatory cytokines.

Variable	IF group	ECG group	LVD group
LFr (n.u.) in 24-hour Holter	0.114 (p = 0.685)	- 0,539 (p = 0.038)*	- 0.075 (p = 0.791)
HFr (n.u.) in 24-hour Holter	0.079 (p = 0.781)	0.539 (p = 0.038)*	0.075 (p = 0.791)
LFr/HFr Ratio in 24-hour Holter	- 0.114 (p = 0.685)	- 0.518 (p = 0.048)*	- 0.075 (p = 0.791)
LFr (n.u.) in postural tilt test (supine position)	0.318 (p = 0.248)	- 0.275 (p = 0.321)	0.339 (p = 0.216)
HFr (n.u.) in postural tilt test (supine position)	- 0.318 (p = 0.248)	0.275 (p = 0.321)	- 0.364 (p = 0.182)
LFr/HFr Ratio in postural tilt test (supine position)	0.318 (p = 0.248)	- 0.293 (p = 0.289)	0.364 (p = 0.182)
LFr (n.u.) in postural tilt test (orthostatic position)	0.100 (p = 0.723)	0.054 (p = 0.850)	0.300 (p = 0.277)
HFr (n.u.) in postural tilt test (orthostatic position)	- 0.100 (p = 0.723)	- 0.054 (p = 0.850)	- 0.300 (p = 0.277)
LFr/HFr Ratio in postural tilt test (orthostatic position)	0.100 (p = 0.723)	0.054 (p = 0.850)	0.304 (p = 0.271)
Interleukin-6	- 0.061 (p = 0.830)	- 0.496 (p = 0.329)	- 0.003 (p = 0.934)
TNF-alpha	0.383 (p = 0.349)	- 0.500 (p = 0.667)	0.429 (p = 397)

* P value < 0,05

HFr: high frequency; LFr: low frequency; n.u.: normalized units; TNF-alpha: Tumor Necrosis Factor-alpha

There were significant correlations between leptin with some ANS assessment indexes in LVD group. Leptin level is associated positively with the LFr component and negatively with the HFr component in 24-hour Holter. No correlations between leptin and inflammatory cytokines were found. The values of correlations of leptin are shown in Table 7.

**Table 7:** Spearman rank correlation coefficients of leptin with autonomic nervous system indices and inflammatory cytokines.

Variable	IF group	ECG group	LVD group
LFr (n.u.) in 24-hour Holter	0,396 (p = 0.143)	- 0,182 (p = 0.869)	0.632 (p = 0.011)*
HFr (n.u.) in 24-hour Holter	- 0.414 (p = 0.125)	- 0.046 (p = 0.516)	- 0.632 (p = 0.011)*
LFr/HFr Ratio in 24-hour Holter	0.414 (p = 0.125)	- 0.034 (p = 0.904)	0.632 (p = 0.011)*
LFr (n.u.) in postural tilt test (supine position)	- 0.236 (p = 0.398)	0.404 (p = 0.136)	0.329 (p = 0.232)
HFr (n.u.) in postural tilt test (supine position)	0.236 (p = 0.398)	- 0.404 (p = 0.136)	- 0.361 (p = 0.187)
LFr/HFr Ratio in postural tilt test (supine position)	- 0.236 (p = 0.398)	0.400 (p = 0.140)	0.361 (p = 0.187)
LFr (n.u.) in postural tilt test (orthostatic position)	0.068 (p = 0.810)	0.200 (p = 0.475)	0.168 (p = 0.550)
HFr (n.u.) in postural tilt test (orthostatic position)	- 0.068 (p = 0.810)	- 0.200 (p = 0.475)	- 0.168 (p = 0.550)
LFr/HFr Ratio in postural tilt test (orthostatic position)	0.068 (p = 0.810)	0.200 (p = 0.475)	0.154 (p = 0.584)
Interleukin-6	0.118 (p = 0.676)	- 0.371 (p = 0.468)	- 0.214 (p = 0.467)
TNF-alpha	0.395 (p = 0.333)	- 0.500 (p = 0.667)	- 0.261 (p = 0.111)

* P value < 0,05

HFr: high frequency; LFr: low frequency; n.u.: normalized units; TNF-alpha: Tumor Necrosis Factor-alpha

## Discussion

We found increased adiponectin levels in patients with Chagas’ heart disease. Furthermore, we observed a relationship in regard to adipocytokine levels and ANS function in Chagas’ heart disease. The levels of adiponectin are associated with reduced sympathetic activity and increased parasympathetic activity and the levels of leptin are associated with increased sympathetic activity and reduced parasympathetic activity in subjects with Chagas’ disease and cardiac involvement.

The levels of adiponectin have already been evaluated in animal models of acute infection with *Trypanosoma cruzi*. In 2005, Coombs et al conducted a study of infected rats during the acute phase of Chagas’ disease, demonstrating that the adipocytes infected with *T*. *cruzi* displayed changes in the secretion of adipocytokines. There were decreased levels of adiponectin and leptin and increased levels of interleukin-6 and TNF-alpha [21]. Another study performed by Nagajyothi et al. showed increased expression of pro-inflammatory cytokines, including interleukin-6 and TNF-alpha in an adipocyte culture infected with *T*. *cruzi*.[22] Ferreira et al. recently observed persistence of *T*. *cruzi* in human adipocytes with chronic Chagas’ cardiomyopathy. However, the data are scarce in relation to adipocytokines levels in the chronic phase of CD in humans.[23]

In heart failure, regardless of the cause, adiponectin has a biphasic behavior, with reduced levels in diastolic HF and increased levels in systolic HF [24]. In our study, patients from IF group had no significant difference in adiponectin levels when compared with control group. This finding reinforces the good prognosis of IF form of Chagas’ disease, similar to general population [4]. We found higher levels of adiponectin in LVD and ECG groups, contrasting with the reduced levels found in the studies using animal models of acute infection [21]. The increase of adiponectin in the LVD group may be explained by the presence of systolic dysfunction and emphasizing that this group had less fat mass, which can also increase the levels of adiponectin.[25,26]

We should however draw attention to the increase of adiponectin in the ECG group. This group did not have a reduction in LVEF or clinical signs of HF and had similar weight, BMI, and fat mass than the IF and control groups. In the REDS-II study, a history of ECG abnormalities in asymptomatic chagasic patients was a prognostic factor for disease progression. The authors observed a moderate rate of progression to cardiomyopathy with systolic dysfunction and HF in patients with ECG abnormalities (1.85% per year) [27].

Although the electrocardiogram is an established marker of severity of Chagas’ disease, the increased adiponectin levels found in patients with ECG abnormalities without systolic dysfunction suggest that adiponectin may help to identify patients at higher risk of developing systolic HF in this group.

It is not well established where adiponectin is predominantly secreted in HF patients and if its increased levels are only a compensation mechanism. Adiponectin is synthetized almost exclusively by adipocytes and its expression seems to be regulated by distinct signaling pathways [12]. Some studies suggest that adiponectin is over expressed in adipocytes from adipose tissue of systolic HF patients [28,29]. Additionally, other studies indicate the presence of a cardiac adiponectin system with independent regulation and with dysfunction in HF. Adiponectin is released from the cardiomyocytes into the peripheral circulation in proportion to extent of systolic dysfunction irrespective of etiologies of HF [30,31]. Moreover, Nagajyothi et al. has shown that adiponectin is considerably produced in the heart during Chagas’ acute infection. However, it is not well established the mechanism by which this occurs and the significance of elevated adiponectin levels in the heart during acute and chronic Chagas’ infection [32].

Many mechanisms have been proposed for the increase of adiponectin in systolic HF of any cause. The patients with cardiac cachexia showed higher adiponectin levels than the patients with systolic HF without cachexia. It is still not clear if the increased adiponectin levels in these patients are a cause or effect of cachexia [25,26]. Bobbert et al. demonstrated that patients with inflammatory cardiomyopathy have higher levels of adiponectin than patients with non-inflammatory cardiomyopathy and that, between the patients with inflammatory cardiomyopathy, those that have higher levels of adiponectin had better outcomes. These data suggest that the increase of adiponectin could act as a compensation mechanism due its anti-inflammatory action [33]. Other mechanisms proposed for the increase of adiponectin in systolic HF are a compensatory effect for the resistance to insulin found in HF or a mechanism of resistance to the action of adiponectin [34].

However, among the variables assessed in our study, the increased levels of adiponectin was associated with ANS function in patients with early phase of Chagas’ heart disease. There was a negative association with the sympathetic branch and a positive association with the parasympathetic branch of ANS in ECG group. Our study does not allow conclusions on casual inference or the effect of one variable over the other. However, previous publications described possible pathways for this association.

Other studies, in different models, demonstrate the same inverse association between adiponectin and sympathetic activity found in our study. Studies in animals and humans, with cold exposure as a way to activate the sympathetic nervous system, showed reduced levels of adiponectin [35,36]. An association has also been reported between hypoadiponectinemia and sympathetic hyperactivity in patients with diabetes mellitus and obstructive sleep apnea [37–40]. This interaction between adiponectin and the sympathetic nervous system seems to be in a two-way. A study carried out by Tanida et al. showed that the intravenous infusion of adiponectin reduced renal sympathetic nerve activity in animal models [41]. On the other hand, *in vitro* and *in vivo* studies conducted using beta-adrenergic agonists have shown decreased expression and secretion of adiponectin through a direct inhibitory effect on the adipocytes [42,43].

Adipose tissue is predominantly innervated by the sympathetic nervous system [44,45]. In our study, we can speculate that the peripheral impairment of the sympathetic nervous system in chagasic patients could lead to a reduction in sympathetic activity in the adipose tissue and increase the secretion of adiponectin. This mechanism can be explained in two ways. The first mechanism is that the impairment of the sympathetic system could lead to the direct loss of the inhibitory effect of the adrenergic tone on the expression and secretion of adiponectin by the adipocytes [42,43]. On the other hand, the main action of the sympathetic nervous system on the adipose tissue is to stimulate lipolysis with increased production of free fatty acids [44,45]. The accumulation of free fatty acids in the adipose tissue reduces the secretion of adiponectin [46,47]. Therefore, the reduction of sympathetic activity in the adipose tissue could reduce lipolysis and reduce the production of free fatty acids, leading to increased levels of adiponectin.

With regard to others metabolic parameters, insulin, in the majority of studies, demonstrate a reduction of its levels or a compromise of its function in Chagas’ disease patients [48–50]. The data on leptin levels from previous studies vary in heart failure patients. Some studies have shown raised levels, especially in more advanced functional classes [17,51], and other studies have demonstrated reduced levels, particularly in patients with cachexia [18,52]. In patients with Chagas’ disease, we have previously found reduced levels in patients with HF when compared with a control group and other forms of CD [19]. In the present study, we did not detect significant differences in the levels of insulin and leptin between the different groups.

However, we observed a positive association between leptin levels and sympathetic activity in LVD group. Leptin’s relationship with the sympathetic component of the ANS is two-way, with increased secretion of leptin leading to increased sympathetic activity, and sympathetic activity inhibiting the synthesis of leptin [53,54]. Paolisso et al. described, in healthy subjects, the same positive association between leptin levels and sympathetic activity found in our study [55]. Other studies demonstrated that acute and chronic increase in plasma leptin levels triggers a remarkable increase in sympathetic activity in various organs including the adipose tissue [56,57].

With regard to associations observed in our study, we have to address that the small sample size, although homogeneous, may have reduced the power to detect other associations.

Our findings in ANS study were also observed in others publications. Autonomic involvement is a well-establishhed feature of CD, in which anatomic and functional abnormalities have been extensively described. The majority of studies observed parasympathetic dysautonomia preceding left ventricular systolic dysfunction and a progressive involvement of sympathetic system in advanced Chagas’ heart disease [7,8,58].

In our study, we observed HFr indexes in LVD group equivalent or higher when compared with control group. We did not interpret these findings as a normal vagal function in patients with systolic dysfunction. The reciprocal relationship or push-pull organization of LFr and HFr components of HRV, which can be better appreciated by using a normalization procedure, shows a view of a predominance of an ANS branch over the other, named as sympatho-vagal balance [20]. So, our study suggests that there was a greater impairment of sympathetic system and a predominance of parasympathetic activity in LVD group, although there was involvement of both. Another reason for this finding is that, for ethical reasons, we chose not to suspend the use of prescribed medications. So all patients in the LVD group were using beta-blockers, which can increase cardiac vagal modulation and lower sympathetic activity, increasing HFr indexes [59].

Another issue to be discussed is the controversy about the use of LFr component as a marker of sympathetic modulation. Some authors consider that this component is a parameter that includes both sympathetic, vagal and baroreflex influences, especially in rest [60]. We used the concept of the international Task Force document of Heart Rate Variability which considers LFr component (especially when expressing it in normalized units) as an index of sympathetic modulation [20].

## Conclusions

In conclusion, we found increased adiponectin levels in Chagas’ heart disease with and without significant systolic dysfunction. Furthermore, adipocytokines levels were associated with some ANS parameters. Adiponectin levels were positively associated with HFr component and inversely associated with LFr component in ECG group and leptin levels were inversely associated with HFr component and positively associated with LFr component of HRV in LVD group.

This is the first study to investigate the influence of cardiac autonomic function, as estimated by HRV, on serum adipocytokines in chagasic patients. We used Chagas’ disease as a model in which autonomic dysfunction influences adipocytokine levels, which are important metabolic parameters in cardiovascular function. A better understanding of the relationship between autonomic and adipose tissue functions can lead to further evaluation for determining which individuals are at the highest risk for cardiovascular disease and mortality, and can also lead to the development of new therapies for reducing that risk.
